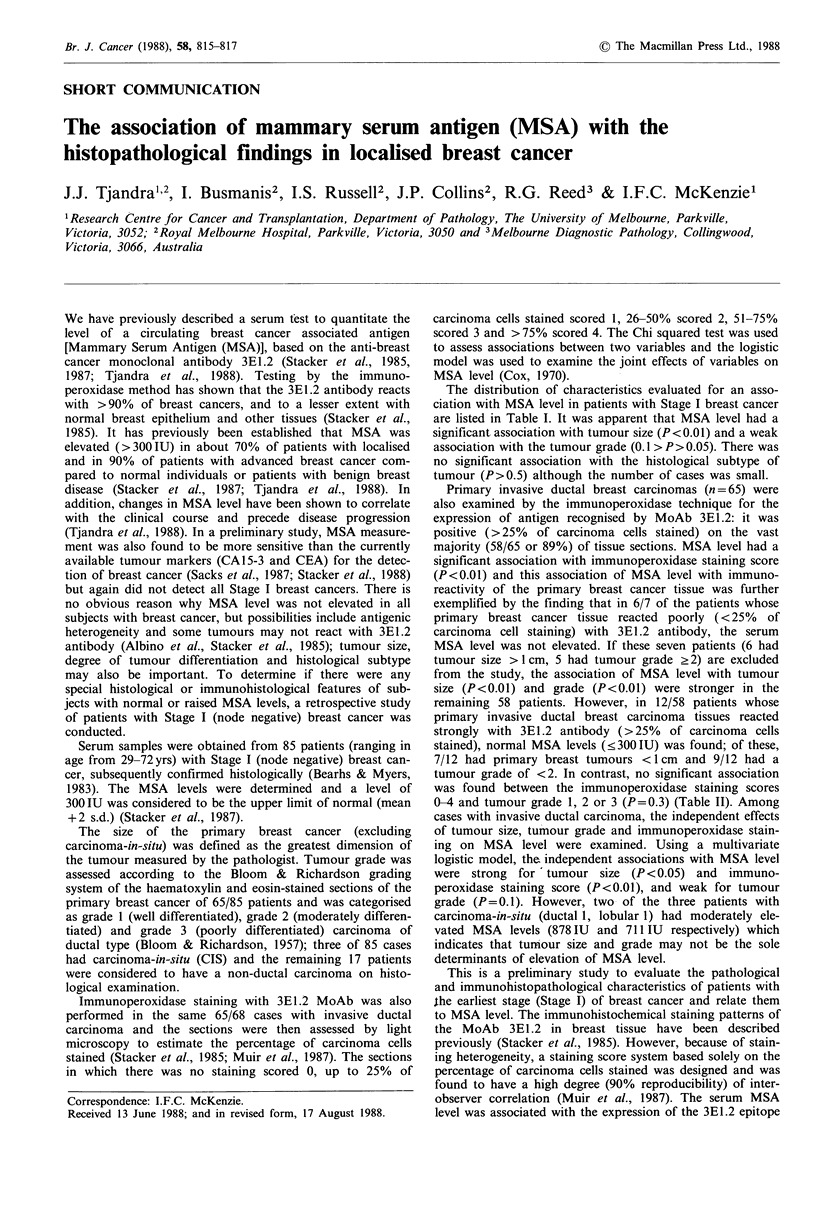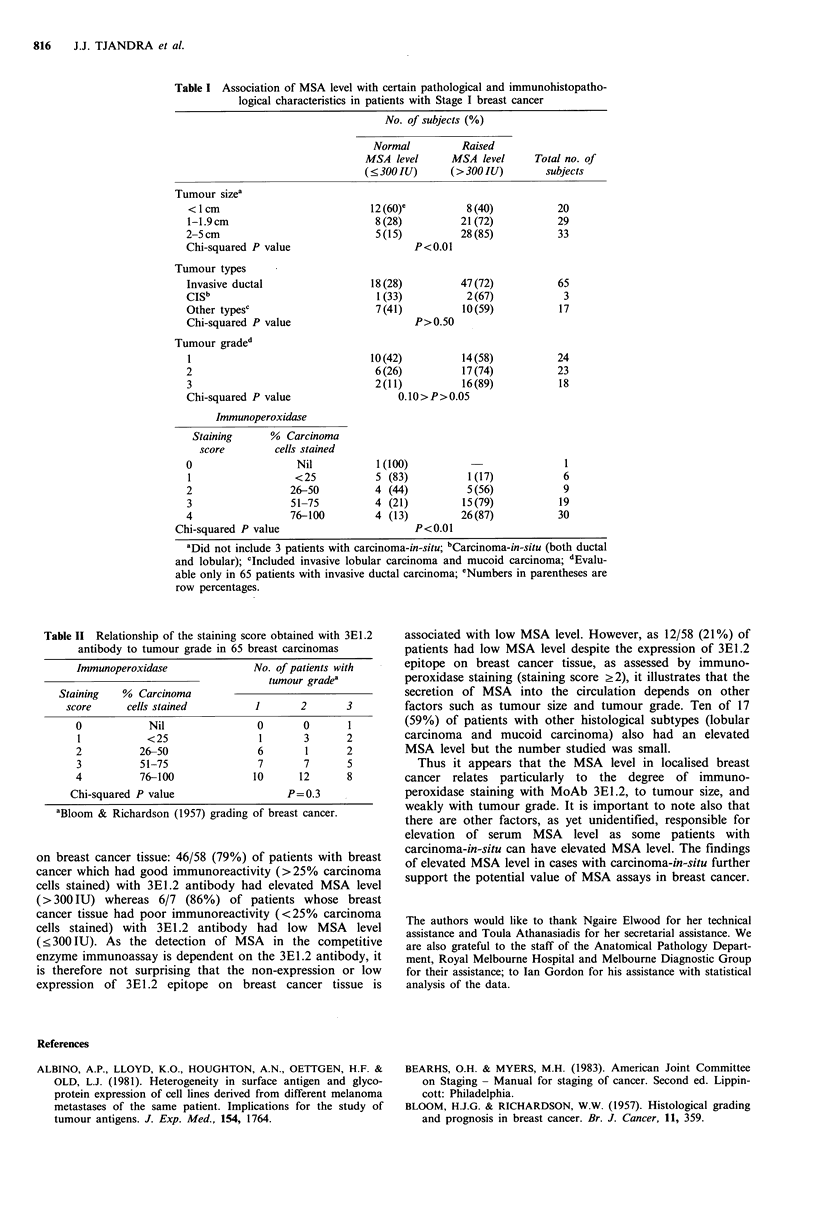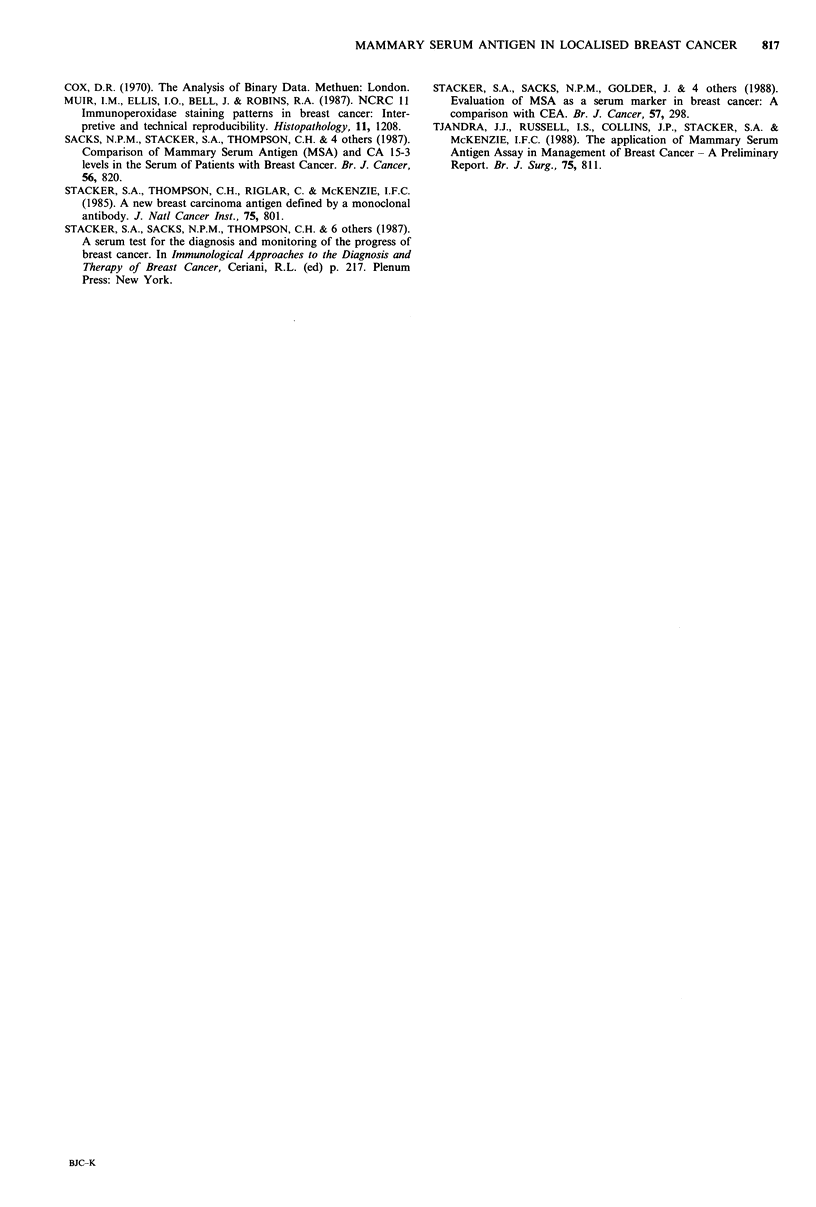# The association of mammary serum antigen (MSA) with the histopathological findings in localised breast cancer.

**DOI:** 10.1038/bjc.1988.316

**Published:** 1988-12

**Authors:** J. J. Tjandra, I. Busmanis, I. S. Russell, J. P. Collins, R. G. Reed, I. F. McKenzie

**Affiliations:** Department of Pathology, University of Melbourne, Parkville, Victoria.


*Br. J. Cancer* (1988), **58**, 815–817 © The Macmillan Press Ltd., 1988

SHORT COMMUNICATION

### The association of mammary serum antigen (MSA) with the histopathological findings in localised breast cancer

J.J. Tjandra[1,2], I. Busmanis[2], I.S. Russell[2], J.P. Collins[2], R.G. Reed[3] & I.F.C. McKenzie[1]

[1]*Research Centre for Cancer and Transplantation, Department of Pathology, The University of Melbourne, Parkville, Victoria, 3052;* [2]*Royal Melbourne Hospital, Parkville, Victoria, 3050 and* [3]*Melbourne Diagnostic Pathology, Collingwood, Victoria, 3066, Australia*

We have previously described a serum test to quantitate the level of a circulating breast cancer associated antigen [Mammary Serum Antigen (MSA)], based on the anti-breast cancer monoclonal antibody 3E1.2 (Stacker *et al.*, 1985, 1987; Tjandra *et al.*, 1988). Testing by the immunoperoxidase method has shown that the 3E1.2 antibody reacts with >90% of breast cancers, and to a lesser extent with normal breast epithelium and other tissues (Stacker *et al.*, 1985). It has previously been established that MSA was elevated (>300 IU) in about 70% of patients with localised and in 90% of patients with advanced breast cancer compared to normal individuals or patients with benign breast disease (Stacker *et al.*, 1987; Tjandra *et al.*, 1988). In addition, changes in MSA level have been shown to correlate with the clinical course and precede disease progression (Tjandra *et al.*, 1988). In a preliminary study, MSA measurement was also found to be more sensitive than the currently available tumour markers (CA15-3 and CEA) for the detection of breast cancer (Sacks *et al.*, 1987; Stacker *et al.*, 1988) but again did not detect all Stage I breast cancers. There is no obvious reason why MSA level was not elevated in all subjects with breast cancer, but possibilities include antigenic heterogeneity and some tumours may not react with 3E1.2 antibody (Albino *et al.*, Stacker *et al.*, 1985); tumour size, degree of tumour differentiation and histological subtype may also be important. To determine if there were any special histological or immunohistological features of subjects with normal or raised MSA levels, a retrospective study of patients with Stage I (node negative) breast cancer was conducted.

Serum samples were obtained from 85 patients (ranging in age from 29–72 yrs) with Stage I (node negative) breast cancer, subsequently confirmed histologically (Bearhs & Myers, 1983). The MSA levels were determined and a level of 300 IU was considered to be the upper limit of normal (mean +2 s.d.) (Stacker *et al.*, 1987).

The size of the primary breast cancer (excluding carcinoma-*in-situ*) was defined as the greatest dimension of the tumour measured by the pathologist. Tumour grade was assessed according to the Bloom & Richardson grading system of the haematoxylin and eosin-stained sections of the primary breast cancer of 65/85 patients and was categorised as grade 1 (well differentiated), grade 2 (moderately differentiated) and grade 3 (poorly differentiated) carcinoma of ductal type (Bloom & Richardson, 1957); three of 85 cases had carcinoma-*in-situ* (CIS) and the remaining 17 patients were considered to have a non-ductal carcinoma on histological examination.

Immunoperoxidase staining with 3E1.2 MoAb was also performed in the same 65/68 cases with invasive ductal carcinoma and the sections were then assessed by light microscopy to estimate the percentage of carcinoma cells stained (Stacker *et al.*, 1985; Muir *et al.*, 1987). The sections in which there was no staining scored 0, up to 25% of carcinoma cells stained scored 1, 26–50% scored 2, 51–75% scored 3 and >75% scored 4. The Chi squared test was used to assess associations between two variables and the logistic model was used to examine the joint effects of variables on MSA level (Cox, 1970).

The distribution of characteristics evaluated for an association with MSA level in patients with Stage I breast cancer are listed in Table I. It was apparent that MSA level had a significant association with tumour size ($P<0.01$) and a weak association with the tumour grade ($0.1>P>0.05$). There was no significant association with the histological subtype of tumour ($P>0.5$) although the number of cases was small.

Primary invasive ductal breast carcinomas ($n=65$) were also examined by the immunoperoxidase technique for the expression of antigen recognised by MoAb 3E1.2: it was positive (>25% of carcinoma cells stained) on the vast majority (58/65 or 89%) of tissue sections. MSA level had a significant association with immunoperoxidase staining score ($P<0.01$) and this association of MSA level with immunoreactivity of the primary breast cancer tissue was further exemplified by the finding that in 6/7 of the patients whose primary breast cancer tissue reacted poorly (<25% of carcinoma cell staining) with 3E1.2 antibody, the serum MSA level was not elevated. If these seven patients (6 had tumour size >1 cm, 5 had tumour grade ≥2) are excluded from the study, the association of MSA level with tumour size ($P<0.01$) and grade ($P<0.01$) were stronger in the remaining 58 patients. However, in 12/58 patients whose primary invasive ductal breast carcinoma tissues reacted strongly with 3E1.2 antibody (>25% of carcinoma cells stained), normal MSA levels (≤300 IU) was found; of these, 7/12 had primary breast tumours <1 cm and 9/12 had a tumour grade of <2. In contrast, no significant association was found between the immunoperoxidase staining scores 0–4 and tumour grade 1, 2 or 3 ($P=0.3$) (Table II). Among cases with invasive ductal carcinoma, the independent effects of tumour size, tumour grade and immunoperoxidase staining on MSA level were examined. Using a multivariate logistic model, the independent associations with MSA level were strong for tumour size ($P<0.05$) and immunoperoxidase staining score ($P<0.01$), and weak for tumour grade ($P=0.1$). However, two of the three patients with carcinoma-*in-situ* (ductal 1, lobular 1) had moderately elevated MSA levels (878 IU and 711 IU respectively) which indicates that tumour size and grade may not be the sole determinants of elevation of MSA level.

This is a preliminary study to evaluate the pathological and immunohistopathological characteristics of patients with the earliest stage (Stage I) of breast cancer and relate them to MSA level. The immunohistochemical staining patterns of the MoAb 3E1.2 in breast tissue have been described previously (Stacker *et al.*, 1985). However, because of staining heterogeneity, a staining score system based solely on the percentage of carcinoma cells stained was designed and was found to have a high degree (90% reproducibility) of interobserver correlation (Muir *et al.*, 1987). The serum MSA level was associated with the expression of the 3E1.2 epitope

Correspondence: I.F.C. McKenzie.
Received 13 June 1988; and in revised form, 17 August 1988.

**Table I** Association of MSA level with certain pathological and immunohistopathological characteristics in patients with Stage I breast cancer

| | | No. of subjects (%) | | |
|---|---|---|---|---|
| | | *Normal MSA level* ($\leq$*300 IU*) | *Raised MSA level* (>*300 IU*) | *Total no. of subjects* |
| Tumour size[a] | | | | |
| <1 cm | | 12 (60)[e] | 8 (40) | 20 |
| 1–1.9 cm | | 8 (28) | 21 (72) | 29 |
| 2–5 cm | | 5 (15) | 28 (85) | 33 |
| Chi-squared *P* value | | *P*<0.01 | | |
| Tumour types | | | | |
| Invasive ductal | | 18 (28) | 47 (72) | 65 |
| CIS[b] | | 1 (33) | 2 (67) | 3 |
| Other types[c] | | 7 (41) | 10 (59) | 17 |
| Chi-squared *P* value | | *P*>0.50 | | |
| Tumour grade[d] | | | | |
| 1 | | 10 (42) | 14 (58) | 24 |
| 2 | | 6 (26) | 17 (74) | 23 |
| 3 | | 2 (11) | 16 (89) | 18 |
| Chi-squared *P* value | | 0.10>*P*>0.05 | | |
| *Immunoperoxidase* | | | | |
| *Staining score* | *% Carcinoma cells stained* | | | |
| 0 | Nil | 1 (100) | — | 1 |
| 1 | <25 | 5 (83) | 1 (17) | 6 |
| 2 | 26–50 | 4 (44) | 5 (56) | 9 |
| 3 | 51–75 | 4 (21) | 15 (79) | 19 |
| 4 | 76–100 | 4 (13) | 26 (87) | 30 |
| Chi-squared *P* value | | *P*<0.01 | | |

[a]Did not include 3 patients with carcinoma-*in-situ*; [b]Carcinoma-*in-situ* (both ductal and lobular); [c]Included invasive lobular carcinoma and mucoid carcinoma; [d]Evaluable only in 65 patients with invasive ductal carcinoma; [e]Numbers in parentheses are row percentages.

**Table II** Relationship of the staining score obtained with 3E1.2 antibody to tumour grade in 65 breast carcinomas

| *Immunoperoxidase* | | *No. of patients with tumour grade*[a] | | |
|---|---|---|---|---|
| *Staining score* | *% Carcinoma cells stained* | *1* | *2* | *3* |
| 0 | Nil | 0 | 0 | 1 |
| 1 | <25 | 1 | 3 | 2 |
| 2 | 26–50 | 6 | 1 | 2 |
| 3 | 51–75 | 7 | 7 | 5 |
| 4 | 76–100 | 10 | 12 | 8 |
| Chi-squared *P* value | | | *P*=0.3 | |

[a]Bloom & Richardson (1957) grading of breast cancer.

on breast cancer tissue: 46/58 (79%) of patients with breast cancer which had good immunoreactivity (>25% carcinoma cells stained) with 3E1.2 antibody had elevated MSA level (>300 IU) whereas 6/7 (86%) of patients whose breast cancer tissue had poor immunoreactivity (<25% carcinoma cells stained) with 3E1.2 antibody had low MSA level ($\leq$300 IU). As the detection of MSA in the competitive enzyme immunoassay is dependent on the 3E1.2 antibody, it is therefore not surprising that the non-expression or low expression of 3E1.2 epitope on breast cancer tissue is associated with low MSA level. However, as 12/58 (21%) of patients had low MSA level despite the expression of 3E1.2 epitope on breast cancer tissue, as assessed by immunoperoxidase staining (staining score $\geq$2), it illustrates that the secretion of MSA into the circulation depends on other factors such as tumour size and tumour grade. Ten of 17 (59%) of patients with other histological subtypes (lobular carcinoma and mucoid carcinoma) also had an elevated MSA level but the number studied was small.

Thus it appears that the MSA level in localised breast cancer relates particularly to the degree of immunoperoxidase staining with MoAb 3E1.2, to tumour size, and weakly with tumour grade. It is important to note also that there are other factors, as yet unidentified, responsible for elevation of serum MSA level as some patients with carcinoma-*in-situ* can have elevated MSA level. The findings of elevated MSA level in cases with carcinoma-*in-situ* further support the potential value of MSA assays in breast cancer.

The authors would like to thank Ngaire Elwood for her technical assistance and Toula Athanasiadis for her secretarial assistance. We are also grateful to the staff of the Anatomical Pathology Department, Royal Melbourne Hospital and Melbourne Diagnostic Group for their assistance; to Ian Gordon for his assistance with statistical analysis of the data.

### References

ALBINO, A.P., LLOYD, K.O., HOUGHTON, A.N., OETTGEN, H.F. & OLD, L.J. (1981). Heterogeneity in surface antigen and glycoprotein expression of cell lines derived from different melanoma metastases of the same patient. Implications for the study of tumour antigens. *J. Exp. Med.*, **154**, 1764.

BEARHS, O.H. & MYERS, M.H. (1983). American Joint Committee on Staging – Manual for staging of cancer. Second ed. Lippincott: Philadelphia.

BLOOM, H.J.G. & RICHARDSON, W.W. (1957). Histological grading and prognosis in breast cancer. *Br. J. Cancer*, **11**, 359.

COX, D.R. (1970). The Analysis of Binary Data. Methuen: London.

MUIR, I.M., ELLIS, I.O., BELL, J. & ROBINS, R.A. (1987). NCRC 11 Immunoperoxidase staining patterns in breast cancer: Interpretive and technical reproducibility. *Histopathology*, **11**, 1208.

SACKS, N.P.M., STACKER, S.A., THOMPSON, C.H. & 4 others (1987). Comparison of Mammary Serum Antigen (MSA) and CA 15-3 levels in the Serum of Patients with Breast Cancer. *Br. J. Cancer*, **56**, 820.

STACKER, S.A., THOMPSON, C.H., RIGLAR, C. & McKENZIE, I.F.C. (1985). A new breast carcinoma antigen defined by a monoclonal antibody. *J. Natl Cancer Inst.*, **75**, 801.

STACKER, S.A., SACKS, N.P.M., THOMPSON, C.H. & 6 others (1987). A serum test for the diagnosis and monitoring of the progress of breast cancer. In *Immunological Approaches to the Diagnosis and Therapy of Breast Cancer*, Ceriani, R.L. (ed) p. 217. Plenum Press: New York.

STACKER, S.A., SACKS, N.P.M., GOLDER, J. & 4 others (1988). Evaluation of MSA as a serum marker in breast cancer: A comparison with CEA. *Br. J. Cancer*, **57**, 298.

TJANDRA, J.J., RUSSELL, I.S., COLLINS, J.P., STACKER, S.A. & McKENZIE, I.F.C. (1988). The application of Mammary Serum Antigen Assay in Management of Breast Cancer – A Preliminary Report. *Br. J. Surg.*, **75**, 811.